# Cytokine expression in the visceral adipose tissue after laparoscopic and conventional surgery in a rodent model

**DOI:** 10.1186/s40001-016-0199-8

**Published:** 2016-02-05

**Authors:** Philipp Lingohr, Jonas Dohmen, Hanno Matthaei, Nils Konieczny, Juliane Hoffmann, Edwin Bölke, Sven Wehner, Jörg C. Kalff

**Affiliations:** Department of General, Visceral, Thoracic and Vascular Surgery, University of Bonn, Sigmund-Freud-Strasse 25, 53127 Bonn, Germany; Department of Radiotherapy and Radiation Oncology, University of Düsseldorf, Moorenstrasse 5, 40225 Düsseldorf, Germany

**Keywords:** Cytokines, Cecum resection, Rats, Appendectomy, Adipose tissue, Laparoscopy, IL-6, Resistin, Leptin

## Abstract

**Background:**

Laparoscopic Surgery has become a worldwide standard procedure for a variety of indications. This has been attributed to a milder postoperative inflammatory response by the innate immune system potentially mediated through immune mediators released by the visceral adipose tissue (VAT). However, an in vivo experimental evidence is lacking and is the issue of our present study.

**Methods:**

Male Wistar rats (N = 24) underwent standardized surgical procedures of conventional cecum resection (CCR), conventional sham operation, laparoscopic cecum resection (LCR), or laparoscopic sham operation. Cytokine expression of leptin, resistin, and IL-6 was analyzed in VAT before and after resection by quantitative RT-PCR.

**Results:**

Postoperative leptin gene expression was reduced in the CCR and LCR groups, while expression was not significantly affected in both sham groups compared to the preoperative levels. In contrast, IL-6 expression was not affected in the LCR group, but was significantly elevated in the CCR cohort. The IL-6 expression was significantly higher in CCR compared to LCR. Resistin expression levels did not differ between all groups.

**Conclusions:**

Our study underlines the role of immunological involvement of VAT in the postoperative phase. Low leptin levels seem to act as a stimulator for energy uptake in order to cope with postoperative stress. A lower IL-6 expression in the LCR compared to the CCR group may indicate a weaker inflammatory activity potentially adding to the clinical benefits observed in patients undergoing LS.

## Background

Laparoscopic procedures have become standard in general surgery with numerous clinical advantages such as a faster postoperative recovery, a shorter hospital stay and reduced postoperative pain. Not surprisingly, also in colorectal surgery a reduction of postoperative morbidity leading to faster recovery has been described [1–3]. Even in less extensive interventions such as appendectomy similar benefits could be verified in favor of the laparoscopic approach [4, 5].

The underlying biology of these apparent advantages is not entirely understood. Thus far, it is known that immunological processes have an essential clinical influence in the postoperative phase. Intestinal motility, for example, has been shown to be substantially impaired by the inflammatory response subsequent to abdominal surgery. As demonstrated in animal experiments [6, 7] as well as in humans [8], these circumstances may lead to severe conditions such as manifest postoperative ileus. We recently showed that differences in postoperative outcomes of LS vs. CS are not caused by the extent of leukocyte influx into the peritoneal cavity [9]. Therefore, we sought to test the hypothesis whether or not the transcriptional output of VAT diverges after open or minimally invasive intestinal surgical interventions [10]. Various peptides secreted by the adipose tissue have a messenger function and mediate information about the local status to several target organs such as the brain, liver, pancreas, immune system, vasculature and muscle [11]. In fact, VAT seems to be highly metabolically active especially in patients with insulin resistance [12]. Interestingly, long-term inflammation of VAT seems to be associated with metabolic disorders such as type 2 diabetes mellitus (T2DM) [13].

In order to further elucidate the immunologic capabilities of VAT, we focus on cytokine expression in this compartment after abdominal surgery. In the second step, we investigate if there is a difference between conventional and laparoscopic surgery in the postoperative phase with respect to cytokines in VAT.

## Methods

### Animals

The present study was approved by the Institutional Review Board (IRB) as well as by the federal animal research committee (LANUV Nordrhein-Westfalen, Recklinghausen, Germany, AZ 84-02-04-2012-A252). The Principles of Laboratory Animal Care (National Academic Press, 2011) were followed. We included 24 male Wistar Han IGS rats with a median body weight of 381 g (range: 270–460 g) provided by Charles River WIGA Deutschland GmbH, Bad Königshofen, Germany. All rats were kept under pathogen-free and standardized conditions (temperature ranging from 20 to 24° C; 12 h of light; 12 h of darkness). We provided free access to food through a standard laboratory diet and assured water supply ad libitum.

### Surgical procedures

Surgery was performed under strictly sterile conditions at the animal operation workplace, House of Experimental Therapy (HET), University of Bonn, Germany. Wistar rats (N = 24) were randomized into four groups: conventional cecum resection group (CCR; N = 6), conventional sham operation group (CSO; N = 6), laparoscopic cecum resection group (LCR; N = 6), and laparoscopic sham operation group (LSO; N = 6). Weight distribution did not differ among those groups. Anesthesia and laparoscopic operations were performed as described previously [14].

Operations were initiated by a subcutaneous injection of 0.03–0.05 mg/kg buprenorphine (Temgesic^®^, Reckitt Benckiser Deutschland GmbH, Mannheim, Germany). Afterwards, rats were fixated in front of a mask and narcotized using isoflurane gas (FORENE^®^, Abbott GmbH & Co. KG, Wiesbaden, Germany), starting with 3 % vol. followed by a reduction to 1.5 % vol. After 2 min, the initial flow of 5 l/min was reduced to 2 l/min till the end of the operation. The anesthesia protocol was identical in all groups.

In all groups (CCR, CSO, LCR, and LSO), VAT was probed through resecting a part of the epididymal fat depot before the intestinal resection was performed (CCR and LCR). This sample served as the preoperative reference specimen.

In the laparoscopic groups (LCR and LSO) a capnoperitoneum (5–7 mmHg) was established using a Veress needle 1 cm subxiphoidally. The needle was replaced by a 3 mm trocar and a 2.7 mm laparoscope (KARL STORZ GmbH & Co. KG, Tuttlingen, Germany) was inserted. Under camera observation an additional 2-mm trocar was placed in the left lower abdomen and a 3 mm trocar in the right lower abdomen. All trocars were fixed using a Polysorb™ 3–0 stay-suture (Covidien Deutschland GmbH, Neustadt/Donau, Germany).

In the LCR and LSO groups, a 3 mm Take-apart^®^ Manhes Bipolar Coagulation Forceps (KARL STORZ GmbH & Co. KG) was inserted in the 3 mm trocar in the right lower abdomen. The epididymal fat depot was carefully identified and a 1.5 cm sample separated with the bipolar coagulation forceps. The forceps were replaced by 3 mm endo-scissors (KARL STORZ GmbH & Co. KG) to resect the VAT specimen. Eventually, it was extracted through the enlarged trocar incision in the right lower abdomen.

In the LCR group, we continued with the cecum resection. Using the 2.7 mm laparoscope as well as a 2 mm endo-grasper (KARL STORZ GmbH & Co. KG), a modified Surgitie™ 2–0 (Covidien Deutschland GmbH) was placed laparoscopically at the proximal cecum and the distal part was coagulated using the bipolar coagulation forceps with 70 mA applied three times for 10 s. in an overlapping manner. Afterwards, the specimen was resected intracorporeally using 3 mm endo-scissors and extracted through the incision in the right lower abdomen.

In the LSO group, rats remained in narcosis without intestinal resection for the same time as the LCR cohort after the VAT was extracted.

Animals of the CCR and CSO group underwent open laparotomy. The preoperative VAT sample was obtained using the bipolar coagulation forceps, as described above, and standard dissecting scissors. In the CCR group, we proceeded with the cecum resection. A modified surgitie™ 2–0 was placed at the proximal cecum and the distal part was coagulated in the same manner as in LCR animals. Afterwards, the specimen was resected using standard dissecting scissors. In the CSO group, the rats remained in narcosis without further manipulation for the same period of time as in CCR group after extraction of the VAT.

All incisions were closed in a two-layer manner with interrupted sutures after surgical procedures.

Operation times differed between 25 and 30 min (mean: 28 min ± 2 min).

**Fig. 1:**
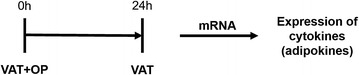
Timeline showing the experimental process

Twenty-four hours after the first operation (sham or intestinal resection), the postoperative VAT sample was retrieved in anesthesia and surgical procedure identical to the method described above (see timeline in Fig. 1).

The twenty-four-hour observation period and the second surgical procedure were unsuspicious for clinical or macroscopic signs of secondary peritonitis. Insufficient stump closure resulting from the cecum resection could not be detected. Afterwards, the animals were sacrificed under sufficient buprenorphine and isoflurane anesthesia by exsanguination.

### RNA Isolation and qRT-PCR

Total RNA from fat samples was extracted with Trizol (Ambion, life technologies, Carlsbad, CA). The RNA concentration and purity were measured with Thermo scientific Nanodrop 2000 spectrophotometer using 1 µl RNA dilute. RNA (200 ng) was reverse-transcribed with High-Capacity cDNA Reverse Transcriptase Kit (Applied Biosystems, Foster City, CA). Taqman system 7900 HT Fast Real-Time PCR System (Applied Biosystems, FosterCity, CA) was used for PCR amplification. Relative gene expression was obtained after normalization to the housekeeping gene Rat GAPD (GAPDH), predesigned by Applied Biosystems (RefSeq: NM_017008.3, Probe Exon Location: 3, Amplicon Size: 87), by the 2-DDCT method, using specific primers for leptin [Qiagen Quantitect Primer Ass Lep(Rat) (NM_013076, XM_008762762)], resistin [Qiagen Rn_Retnlb_1_SG QuantiTect Primer Assay (NM_181625, XM_006248282)], and IL-6 [Applied Biosystems IL6rev Primer Rat (5‘AAG GCA ACT GGC TGG TCT-‘3)].

### Statistics

Statistical analyses were performed using GraphPad Prism^®^ 4 (GraphPad Software, Inc., San Diego, California). Comparison between groups was carried out using analysis of variance (ANOVA) and a post hoc *t* test. A *p* value <0.05 was considered as statistically significant.

## Results and discussion

The aim of our experimental study was the meticulous assessment of extent and pattern of postoperative cytokine expression occurring in VAT. Hence, we measured the following key cytokines, which are well known to be secreted by adipose tissue.

### Leptin

Leptin is a 16 kDa protein secreted mainly by the adipose tissue into the blood circulation. It acts by binding to obesity receptors (ObR), which are widely expressed in the hypothalamus and cerebellum [15]. Leptin has a critical regulatory function in energy storage. Full energy storages and food intake increase the level of circulating leptin [16]. In turn, high circulating leptin normalizes the metabolic status again by lowering the food intake for eventual body weight reduction [17]. However, in most overweight individuals plasma levels of leptin are elevated, which suggests that obesity may be associated with leptin resistance [18].

Furthermore, leptin is described as a proinflammatory cytokine [19] which is suspected to be involved in acute and chronic inflammatory metabolic disorders [20]. Its deficiency, however, increases the risk of infections and is linked with dysbalance of other cytokines [21]. Interestingly, obese subjects in fact have elevated leptin blood levels contributing to a chronic proinflammatory state [22], while a beneficial role of this adipokine in the regulation of acute immune function during sepsis has been evidenced [23].

**Fig. 2:**
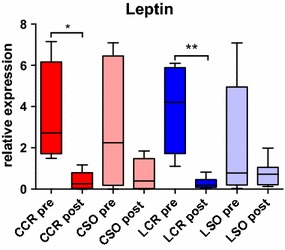
Leptin expression. Pre- and postoperative leptin expression in VAT with a significantly decreased postoperative expression in the CCR (*p < 0.05) and LCR (**p < 0.01) group

In our model, we examined the leptin expression in normal weight rodents in the acute postoperative phase. We found a significant decrease of leptin expression in VAT 24 h after cecum resection in CCR and LCR animals compared to the preoperative levels. In sham operated animals decrease was not significant (Fig. 2).

Other studies however found an increase of serum leptin during bacterial infection [24], acute inflammatory conditions [25] and after surgical stress [26, 27]. Leptin seems to stimulate the proliferation and activation of human monocytes [28, 29] and enhance the synthesis of proinflammatory cytokines by macrophages [30].

Frederich et al. [31] as well Klok et al. [16] stated that leptin is released by energy storages with high serum leptin levels indicating full storages thereby acting as an adipostat. Following this hypothesis, a decrease of leptin 24 h after cecum resection in our study may be regarded as an immunologic mechanism to stimulate essential energy uptake to cope with the posttraumatic inflammatory process.

### IL-6

**Fig. 3:**
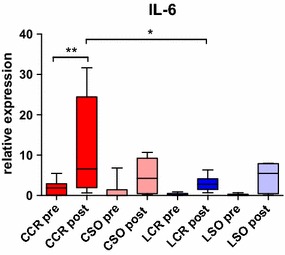
IL-6 expression. Pre- and postoperative IL-6 expression in VAT with a significantly increased postoperative expression in the CCR group (**p < 0.01) also compared to the LCR group (*p < 0.05)

IL-6 is a proinflammatory cytokine with multiple regulatory effects during inflammation mediated through soluble and membrane-bound IL-6 receptors [32]. It has been suggested that IL-6 is a key protein to induce chronic metabolic inflammation in adipose tissue [33]. Systemic IL-6 has been observed immediately after major surgical interventions, while high levels proved to correlate with an increased risk of complications [8]. Paralleling these observations, we measured increased IL-6 expression in VAT samples in the CCR group (Fig. 3), while LCR and sham animals had normal values. These results might be a sign of VAT involvement in the immediate postoperative inflammatory metabolism. However, it remains unclear if peritoneal macrophages, attached to the serosal surface of the VAT, or resident VAT macrophages contribute to IL-6 production. However, since CCR animals had a significantly higher IL-6 expression in VAT 24 h after surgery compared to LCR, our data indicate that minimally invasive procedures result in a milder inflammatory response.

### Resistin

**Fig. 4:**
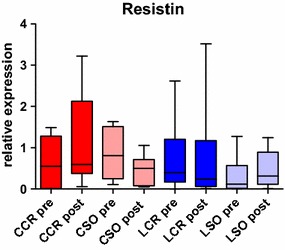
Resistin expression. Pre- and postoperative resistin expression in VAT with no significant changes in any group

Resistin is an adipose tissue-secreted hormone which is also linked to the complex role of adipocyte-derived hormones in the immune system and during inflammation [34]. For example, an increased expression of resistin in subcutaneous and human epicardial adipose tissue was detected subsequent to cardiac surgery [35]. We did not find a significant change in resistin expression in VAT among our groups (Fig. 4) which is an argument against a metabolic involvement of the VAT during the postoperative phase in general. The significant changes in leptin expression (see above) can therefore rather be interpreted as an immunologic than a metabolic regulation mechanism of the VAT to provide more energy resources.

Some relevant limitations of our study are that we did not show the alteration of the protein levels of leptin, IL-6, and resistin and the small numbers of animals hampering statistically strong conclusions.

## Conclusions

In conclusion, we present data of postoperative cytokine expression levels in VAT including leptin, IL-6, and resistin. The postoperative reduction of leptin could indicate an immunologic contribution of this cytokine in posttraumatic inflammatory processes. Low leptin levels thereby seem to act as an immunologic regulation mechanism to provide the energy needed to manage the posttraumatic inflammatory process as there is a decrease in all groups after manipulation.

The postoperative IL-6 levels were significantly increased in the CCR group compared to the LCR group. This might be one factor explaining the oftentimes favorable postoperative course observed in patients undergoing minimally invasive operations.

## Abbreviations

CCR: conventional cecum resection
CS: conventional surgery
CSO: conventional sham operation
LCR: laparoscopic cecum resection
LS: laparoscopic surgery
LSO: laparoscopic sham operation
VAT: visceral adipose tissue
vs.: versus
